# Takotsubo Cardiomyopathy in a Patient with Undiscovered Sigmoid Colon Cancer

**DOI:** 10.1155/2017/4563203

**Published:** 2017-03-09

**Authors:** Huang Po-Yen, Ku Po-Ming

**Affiliations:** Department of Cardiology, Chi-Mei Medical Center, Liuoying, Taiwan

## Abstract

Takotsubo cardiomyopathy (TTC) is a stress-related cardiomyopathy that is characterized by reversible left systolic dysfunction, which appears to be precipitated by sudden emotional or physical stress in the absence of myocardial infarction. Here we present a rare case that clinically presented with intermittent abdominal pain, initially impressed as non-ST elevation myocardial infarction and congestive heart failure but with a normal coronary angiogram. Her symptoms relieved spontaneously without returning. Sigmoid colon cancer was diagnosed via colonoscopy later due to persistent abdominal discomfort. In the absence of detectable emotional or physical stress factors, the newly diagnosed sigmoid colon cancer was the only possible trigger factor of TTC. We offer this case as a reminder that cancer should be considered in the differential diagnosis of patients presenting with the etiology of TTC.

## 1. Introduction

Takotsubo cardiomyopathy (TTC) is characterized by reversible left systolic dysfunction, which appears to be precipitated by sudden emotional or physical stress in the absence of myocardial infarction. It is also known as “stress-related cardiomyopathy,” “left ventricular apical ballooning,” “ampulla cardiomyopathy,” and “broken heart syndrome.” The most commonly used criteria were proposed by the Mayo Clinic and were revised in 2008 [1]. Patients must satisfy all criteria, including the following: (1) transient hypokinesis, akinesis, or dyskinesis in the left ventricular mid-segments with or without apical involvement; regional wall motion abnormalities that extend beyond a single epicardial vascular distribution; and, frequently but not always, a stressful trigger; (2) the absence of obstructive coronary disease or angiographic evidence of acute plaque rupture; (3) new electrocardiogram (ECG) abnormalities or modest elevation in cardiac troponin; and (4) the absence of pheochromocytoma or myocarditis. Although cancer patients have many comorbidities, TTC is rare among cancer patients. On the other hand, cancer may play a role as a trigger of TTC.

## 2. Case Report

A 58-year-old Taiwanese woman was brought to the emergency department (ED) due to intermittent epigastric pain that had lasted for weeks but became exacerbated in the previous few hours. Intermittent lower abdominal pain during defecation with tenesmus was also noted for about 1 month. Other relative symptoms included mild dyspnea on exertion, diaphoresis, hiccupping, and vomiting, which became worse in recent days. She had no past medical history of hypertension or diabetes mellitus. She also had neither known family history nor a history of smoking or drug abuse. There was no postural nocturnal dyspnea, palpitation, or radiation pain. She had no body weight changes in the past 6 months.

**Figure 1:**
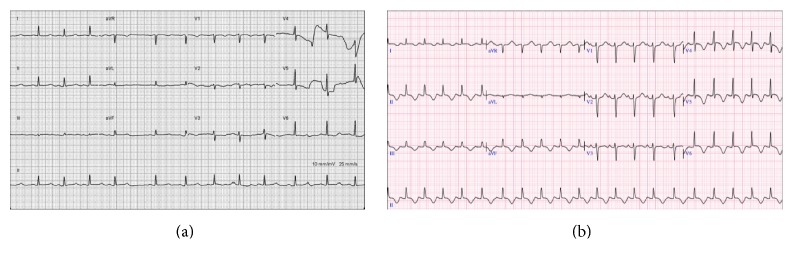
(a) Initial electrocardiogram (ECG) of the patient at the emergency department (ED). (b) Follow-up ECG at the ward 24 hours later.

**Figure 2:**
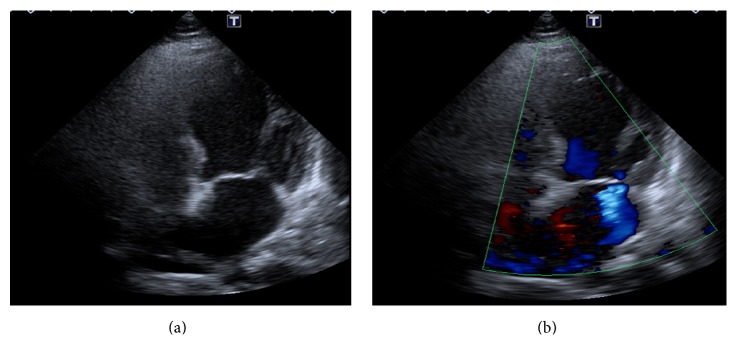
(a) Left ventricle apical hypokinesis during the systolic phase. (b) Mild to moderate mitral valve regurgitation was noted.

**Figure 3:**
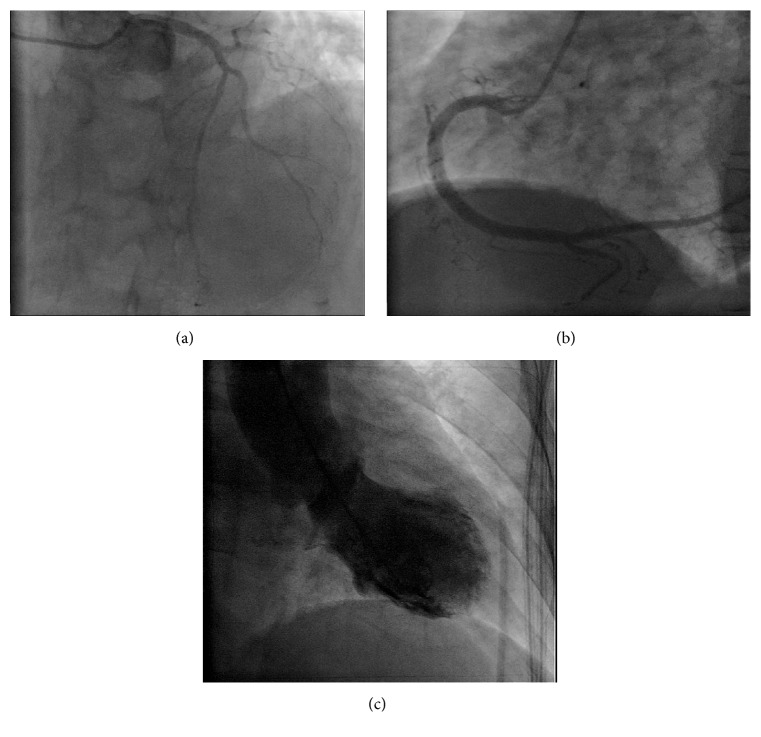
(a), (b) Patent left and right coronary arteries and (c) typical apical ballooning of the left ventricle at the systolic phase.

A physical examination at the ED revealed a regular heart rate at 75 beats per min, a blood pressure of 154/101 mmHg, a grade 2 systolic heart murmur at the apex, and clear breathing sounds on auscultation, mild tenderness at the epigastric area, and no edema of the limbs. An initial ECG showed a premature atrial contraction and insignificant ST-T changes (Figure 1(a)). Initial laboratory tests showed mild leukocytosis (WBC: 10000/*μ*L, segment: 84.1%), Hb 14.4 g/dL, platelet count 163000/*μ*L, glutamic pyruvic transaminase (GPT) 22 IU/L, creatinine 1.0 mg/dL, troponin I 0.06 ng/mL, and creatinine kinase MB 0.8/ng/Ml. After fluid hydration, the patient was admitted to the gastrointestinal ward for suspected peptic ulcer disease. At the ward, a pan-endoscopy showed only mild erythematous gastritis, and the procedure went smoothly without obvious discomforts. However, her epigastric pain seemed stronger and she developed palpitation, diaphoresis, and vomiting at the ward on the following day. Due to the exacerbated symptoms at the ward, a second ECG was performed, which showed new diffused T wave inversion over leads II, III, aVF, and V4–6 (Figure 1(b)). A follow-up laboratory test showed elevated cardiac enzymes: troponin I 2.26 ng/mL (normal < 0.05; 0.06 ng/mL at ED) and creatinine kinase MB 10.3 ng/mL (normal < 3.6; 0.8 ng/mL at ED). A cardiovascular section was then assessed. A transthoracic echocardiogram revealed mild to moderate mitral regurgitation and anterior-apical wall hypokinesis with ejection fraction of 41% (Figures 2(a) and 2(b)). The patient was then transferred to the intensive care unit, and cardiac catheterization was arranged for suspected non-ST elevation myocardial infarction. A coronary angiography showed patent coronary arteries (Figures 3(a) and 3(b)) and a left ventriculography showed apical ballooning of the left ventricle, compatible with TTC (Figure 3(c)). The symptoms improved after several days' treatment with diuretics (furosemide, 20 mg twice per day) and propranolol (10 mg, twice per day).

**Figure 4:**
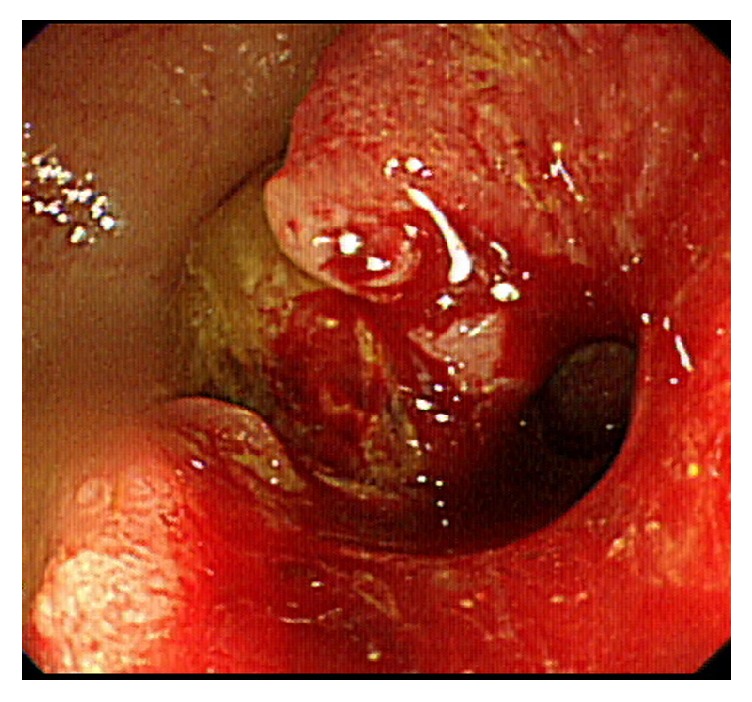
A circumscribed mass with near total obstruction of the sigmoid colon.

In searching for her triggering factors, there were no detectable emotional or physical factors, including the results of an endocrine survey. The patient was then discharged a few days later with improved symptoms. At OPD, a colonoscopy was further arranged due to intermittent lower abdominal pain during defecation, which accidentally revealed a circumscribed mass with near total obstruction of the sigmoid colon about 20 cm from the anal verge (Figure 4). Her tumor markers were within the normal range. The patient was then referred to a surgeon for laparoscopic radical sigmoidectomy, and the biopsy showed adenocarcinoma of the sigmoid colon (cT3N0M0). A follow-up ECG 3 months later revealed an improved ejection fraction of 70% without regional wall motion abnormalities or significant mitral regurgitation. No recurrent angina or dyspnea was present, and the patient is currently being followed up at the oncology OPD.

## 3. Discussion

It is hard to distinguish TTC from acute coronary syndrome in the early clinical stages. Nearly 90% of reported cases of stress-induced cardiomyopathy occurred in postmenopausal women [2]. TTC may account for up to 2% of overall acute coronary syndrome cases and has a low mortality rate of less than 2% and a recovery rate greater than 95% [3, 4]. The etiology of TTC is thought to be related to stress and catecholamine-induced myocardial injury with elevated norepinephrine levels noted in many patients [5]. About 44% of the stressful events that initiate TTC are emotional in nature, and 36% are physical [6].

In the case described here, the persistent epigastric and lower abdominal symptoms lasted for weeks and eventually developed into dyspnea after left ventricular dysfunction proceeded. It is possible that the TTC was induced by pan-endoscopy [7], but the procedure went smoothly and did not induce any intensive pain or vomiting. Atypical angina and dyspnea already occurred before the invasive examination. Procedure-related stress cardiomyopathy cannot be excluded but the probability is low. The patient presented with LV apical hypokinesis both on ECG and ventriculography with transient mitral regurgitation, which has been reported before [8–10].

Among TTC cases overall, cancer has been rarely reported. Among the cancer-related TTC cases, lymphoproliferative disorder (30%), gastrointestinal cancer (15%), and lung cancer (12.5%) were among the most common types observed [11]. Among these patients, perisurgical procedures (such as abdominal surgery or stem cell transplant) were the most common trigger factors (37.5%), followed by chemotherapeutic agents use (13.16%). Emotional stress related to both cancer diagnosis and treatment was also likely a contributor, as patients diagnosed with cancer have been shown to have significantly higher rates of anxiety and depression [12]. Other stressors include changes in their home and family life and fear of disease or death, and those with chronic psychological stress are at risk of developing TTC, which is then triggered by an acute stressor [13]. There have been rare case reports of TTC noted before a diagnosed cancer history. Burgdorf and colleagues have reported a 23.6% incidence of cancer in a cohort of 191 patients with TTC [14], and about 14% new diagnosed malignancy rate in TTC group after a 3-year follow-up (the rate in the control group was zero). This suggests that malignancy may contribute to the pathophysiology of TTC. In some hypotheses, the effects of catecholamines on myocardial function may be induced by a switch in the *β*2-adrenergic receptor (*β*2-AR) intracellular signalling from a Gs to Gi protein [15], which further increases the level of inflammatory mediators. Inflammation may lead to activation of p38 mitogen-activated protein kinase, a downstream effector of the *β*2-AR signalling pathway that has a negative inotropic effect [16–18]. The relationship between malignancy, inflammation, and myocardial damage still requires further investigation. In conclusion, malignancy screening should be considered if available in patients with unknown cause of TTC.
